# Supplementary material for: Preferred versus Actual Place of Care and Factors Associated with Home Discharge among Korean Patients with Advanced Cancer: A Retrospective Cohort Study
Source: Healthcare (Basel). 2023 Jul 5;11(13):1939. doi: 10.3390/healthcare11131939 (PMC10341285; doi:10.3390/healthcare11131939)
Supplement: Supplementary file 1 [file healthcare-11-01939-s001.zip › healthcare-2389173-supplementary.pdf]

Supplementary Table S1. Characteristics of patients who preferred home as place of care by the actual discharge place

Variables	Total (N=210)		Discharged home (N=113)		Discharged to hospital (N=48)		Death (N=49)		*P* value
	N	%	N	%	N	%	N	%	
Age									0.07
median (IQR)	64 (56.2-73)		64 (53-73)		63 (54-73)		69 (63-74)		0.06 [a]
<45	20	9.5	13	11.5	7	14.6	0	0	
≥45, <65	87	41.4	49	43.4	18	37.5	20	40.8	
≥65	103	49	51	45.1	23	47.9	29	59.2	
Gender									0.04
Male	122	58.1	58	51.3	29	60.4	35	71.4	
Female	88	41.9	55	55	19	39.6	14	28.6	
Residence									0.85 [b]
Metropolitan	175	83.3	94	94	38	79.2	43	87.8	
Urban	19	9.0	10	10	6	12.5	3	6.1	
Suburban/rural	16	7.0	9	9	4	8.3	3	6.1	
Medical insurance									0.48 [b]
National health insurance	201	95.7	106	93.8	47	97.9	48	97.9	
Medical aid	6	2.9	5	4.4	1	2.1	0	0	
Other	3	1.4	2	1.8	0	0	1	2.1	
Marital status									0.32 [b]
Married	162	77.5	86	76.1	34	70.8	42	87.5	
Divorced	10	4.8	5	4.4	3	6.2	2	4.2	
Bereaved	17	8.1	8	7.1	5	10.4	4	8.3	
Single	16	7.7	10	8.8	6	12.5	0	0	
Unknown	4	1.9	4	3.5	0	0	0	0.	
Religion									0.11

Christian	52	24.9	29	25.7	17	35.4	6	12.2	
Catholic	28	13.4	16	14.2	2	4.2	10	20.8	
Buddhism	35	16.7	20	17.7	9	18.8	6	12.5	
Other	3	1.4	2	1.8	0	0	1	2.1	
None	78	37.3	38	33.6	19	39.6	21	43.8	
Unknown	13	6.2	8	7.1	1	2.1	4	8.3	
Caregiving									
No. of families living together									0.12
0	4	1.9	0	0	3	6.2	1	2.1	
1	94	44.7	49	43.4	26	54.2	19	39.6	
2 or more	92	43.8	50	44.2	17	35.5	25	52.1	
Unknown	19	9.0	14	12.4	2	4.2	3	6.2	
Relationship of main caregiver									0.20
Spouse	129	62	76	66.4	24	51.5	30	62.5	
Son/daughter	28	13.5	17	15.0	5	10.6	6	12.5	
Parents	41	19.7	17	15.0	14	29.8	10	20.8	
≥2nd degree relatives	6	2.9	2	1.8	3	6.4	1		
Other	1	0.5	1	0.9	1	2.1	0	0	
Unknown	3	1.4	1	0.9	1	2.1	1	2.1	
Type of cancer [c]									0.50 [b]
Lung	42	20	23	20.4	7	14.6	12	24.5	
Breast	11	5.2	7	6.2	3	6.2	1	2.0	
Gastrointestinal	36	17.1	20	17.7	9	18.8	7	14.3	
Hepatobiliary-pancreas	53	25.2	24	21.2	11	22.9	18	36.7	
Hematologic malignancy	16	7.6	11	9.7	3	6.2	2	4.1	
Other	52	24.8	28	24.8	15	31.2	9	18.4	
Treatment status									<0.001
On chemotherapy	77	37	55	48.7	16	34.0	6	12.5	
Off chemotherapy	131	63	58	51.3	31	66.0	42	87.5	

Route of admission									0.06
via outpatient clinic	82	39	52	46.0	13	27.1	17	34.7	
via emergency department	128	61	61	54.0	35	72.9	32	65.3	
Duration of admission									
median (IQR)	15 (9-28)		9 (6-14.5)		18.5 (3.2-30.8)		23 (13-38)		0.04 [a]
Major medical problem									
Confusion	18	8.7	7	6.2	5	10.4	6	12.8	0.24 [b]
Pain	153	73.6	8	71.4	35	72.9	38	79.2	0.64
Fever	35	16.8	16	14.2	12	25.0	7	14.9	0.28 [b]
Dyspnea	74	35.4	3	27.4	15	31.2	28	58.3	0.01
Nausea/vomiting	36	17.2	18	15.9	10	20.8	8	16.7	0.75 [b]
Poor oral intake	111	53.1	47	41.6	27	56.2	37	77.1	<0.001
Urologic symptoms	10	4.8	6	5.4	0	0	4	8.3	0.16 [b]
Bleeding (any)	11	5.3	5	4.4	3	6.2	3	6.2	0.85 [b]
MDR pathogen	12	5.7	3	2.7	3	6.2	8	16.7	0.55 [b]

Abbreviations: IQR, interquartile range; MDR, multidrug resistant

[a] Mann-Whitney U test

[b] Fisher's exact test

[c] Others include malignant neoplasms of lip, oral cavity and pharynx, thyroid and other endocrine glands, eye, brain and other parts of central nervous system, skin, mesothelial and soft tissue, male, female genital organs, urinary tract, ill-defined, other secondary and unspecified sites.

Supplementary Table S2. Factors associated with unintended hospital visits within 2 months after discharge

Variables	With unintended hospital visits within 2 months (N=37)		Without unintended hospital visits within 2 months (N=76)		*P* value
	N	%	N	%	
Age					
median (IQR)	63.5 (53-74.2)		64 (54-72)		0.19 [a]
<45	5	13.5	8	10.5	0.69
≥45, <65	14	37.8	35	46.1	
≥65	18	48.6	33	43.4	
Gender					1
Male	19	51.4	39	51.3	
Female	18	48.6	37	48.7	
Type of cancer [b]					0.69 [c]
Lung	5	13.5	18	23.7	
Breast	3	8.1	4	5.3	
Gastrointestinal	7	18.9	13	17.1	
Hepatobiliary-pancreas	9	24.3	15	19.7	
Hematologic malignancy	5	13.5	6	7,9	
Other	8	21.6	20	26.3	
Treatment status					0.01
On chemotherapy	25	67.6	30	39.5	
Off chemotherapy	12	32.4	46	60.5	
Route of admission					0.83
via outpatient clinic	16	43.2	36	47.4	
via emergency department	21	56.8	40	52.6	
Major medical problem at the time of discharge					
Confusion	0	0	1	1.3	1 [c]
Pain	25	67.6	54	72.0	0.79
Opioid use	24	64.8	52	68.4	0.87
Dose of opioid (morphine equivalent dose, median(IQR)	20 (0-91.7)		34.6 (0-90.2)		0.79 [b]
Fever	4	5.6	1	2.8	0.87 [c]
Dyspnea	15	19.7	3	8.1	0.19
Nausea/vomiting	4	5.3	2	5.4	1 [c]
Poor oral intake	20	26.3	16	43.2	0.11
Requirement of parenteral nutrition(any)	10	27.0	11	15	0.22 [c]
Requirement of parenteral nutrition(only)	3	8.1	4	5.3	0.68 [c]
Urologic symptoms	3	8.1	3	3.9	0.63 [c]
Bleeding (any)	0	0.0	1	1.3	1 [c]
MDR pathogen	1	2.7	2	2.6	1 [c]

Abbreviations: IQR, interquartile range; MDR, multidrug resistant

[a] Mann-Whitney U test

[b] Others include malignant neoplasms of lip, oral cavity and pharynx, thyroid and other endocrine glands, eye, brain and other parts of central nervous system, skin, mesothelial and soft tissue, male, female genital organs, urinary tract, ill-defined, other secondary and unspecified sites.
[c] Fisher's exact test
